# Glucocorticoid and inflammatory reactivity to a repeated physiological stressor in insomnia disorder^☆^

**DOI:** 10.1016/j.nbscr.2018.06.001

**Published:** 2018-06-20

**Authors:** J.K. Devine, S.M. Bertisch, H. Yang, J. Scott-Sutherland, A. Wilkins, V. Molina, K. Henrikson, M. Haack

**Affiliations:** a Beth Israel Deaconess Medical Center and Harvard Medical School, 330, Brookline Ave., Boston, MA 02215, USA; b University of New England College of Osteopathic Medicine, 11 Hills Beach Rd, Biddeford, ME 04005, USA

**Keywords:** Insomnia disorder, Stress reactivity, Cold pressor test, HPA, Inflammation, GC sensitivity

## Abstract

Despite known associations of insomnia disorder with alterations in cytokine and glucocorticoid (GC) production, neither the sensitivity of immune cells to a GC signal nor the reactivity of the hypothalamus-pituitary-adrenal (HPA) axis and inflammatory system to stress, or adaptation of these systems to repeated stress have been assessed in patients with insomnia. To investigate potential dysregulation in stress reactivity and adaptation to repeated exposure, a physiological stressor (the cold pressor test; CPT) was repeatedly administered to N = 20 participants with insomnia disorder (based on DSM-V, 18 females, age 30 ± 2.5 years) and N = 20 sex-matched healthy controls following an at-home actigraphy and in-laboratory PSG. HPA and inflammatory markers (serum cortisol, plasma interleukin [IL]-6) were measured at baseline/resting levels and following each of the three CPTs. In addition, sensitivity of monocytes to the synthetic GC dexamethasone was assessed in-vitro at baseline levels in order to examine the cortisol-IL-6 interplay at the cell level. Compared to healthy controls, individuals with insomnia disorder exhibited shorter sleep duration as assessed by actigraphy and PSG (p ≤ 0.05). HPA, but not inflammatory reactivity to the repeated CPT challenge was greater in insomnia disorder (p ≤ 0.05 for group effect), due to greater cortisol responses to the initial CPT (p ≤ 0.05). There were no between-group differences in the ability of the HPA to adapt to stress repetition nor in basal/resting levels of cortisol, IL-6, and GC sensitivity. These findings suggest that insomnia disorder potentiates HPA axis reactivity to initial/novel stressors, which may constitute a pathway underlying adverse health consequences in the long term.

## Highlights

• Insomnia disorder potentiates HPA, but not inflammatory reactivity to a novel stressor.
• The ability of the HPA axis to adapt to the repeated exposure to the same stressor is unchanged in insomnia.
• Basal/resting levels of cortisol, IL-6, and their interplay at cell level (i.e., GC sensitivity of monocytes) are unchanged in insomnia.

## Graphical abstract

**Figure:**
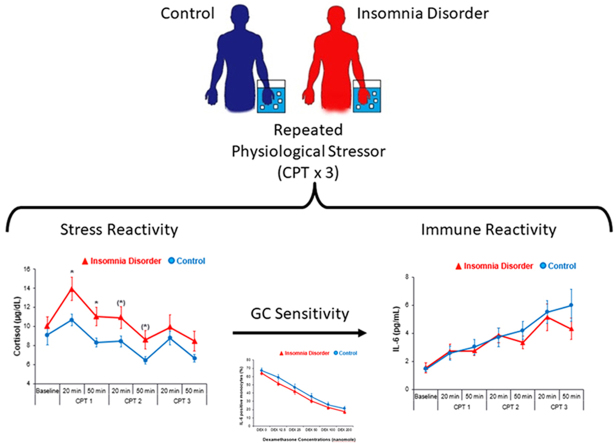

## 1 Introduction

Insomnia disorder is highly prevalent, frequently comorbid with a variety of mental and medical conditions, and significantly impacts psychological well-being and physical health (Pigeon, 2010). It has been hypothesized that insomnia symptomology may be due to physiological hyperarousal of multiple systems, including the hypothalamic-pituitary-adrenal (HPA) axis and inflammatory system (Bonnet and Arand, 2010, Riemann, 2010). However, the relationship between insomnia and alterations in the HPA axis and inflammatory system has yet to be determined (Balbo et al., 2010, Irwin et al., 2016, Riemann, 2010) as current findings are inconsistent. For example, insomnia is related to alterations in basal inflammatory markers, as indicated by findings of increased levels of interleukin-6 (IL-6) in some studies (Burgos et al., 2006, Vgontzas and Chrousos, 2002), but not in other studies (Floam et al., 2015, see also comprehensive review on sleep disturbances by Irwin et al. 2016). Levels of the glucocorticoid cortisol, an HPA axis marker, have been found to be increased in insomnia in some studies (Floam et al., 2015, Rodenbeck et al., 2002, Vgontzas et al., 2001), but not others (Riemann et al., 2002, Varkevisser et al., 2005; see also review by Balbo et al. 2010).

Beyond basal/resting activity of the HPA axis and inflammatory system, their reactivity in response to stressors and challenges could potentially be altered in insomnia as well (Buckley and Schatzberg, 2005, Meerlo et al., 2008). In support of this concept, poorer sleep quality was recently associated with greater IL-6 reactivity following a psychosocial stressor in postmenopausal women (Prather et al., 2014). In accordance, IL-6 reactivity following a series of cognitive challenges was higher in men and postmenopausal women age 50 and older who reported poor sleep quality compared to those with good sleep quality (Heffner et al., 2012). Further, it was recently found that poor sleep quality in otherwise healthy individuals was associated with greater cortisol reactivity in response to a physiological stress challenge, namely, the cold pressor test (CPT) (Goodin et al., 2012). The CPT involves the immersion of the hand in ice-cold water kept at about 3 degree C for up to three minutes. It is one of the most commonly-used laboratory physiological challenge tests, provoking not only unpleasantness, but also increases in sympathetic nervous system and HPA axis activity (Al’Absi et al., 2002, McRae et al., 2006), as well as inflammatory markers, including IL-6 (Edwards et al., 2009, Griffis et al., 2013). Considering that most individuals have to deal with stressful challenges on a daily basis, failure of the HPA axis and inflammatory system to habituate to daily challenges may elevate disease risk in the long term (Grissom and Bhatnagar, 2009). Habituation, i.e., a decreased response across the repeated exposure to the same stressful challenge, is a key feature of the adaptive nature of many biological systems, and has been observed in response to a variety of psychological and physiological stressors (Grissom and Bhatnagar, 2009). To our knowledge, no studies have measured whether insomnia may affect the ability of the HPA axis or inflammatory system to adapt to repeated stressful challenges. This is a novel aspect in understanding the pathophysiology of insomnia disorder.

The first aim of this study was to explore whether measures that capture the reactivity and adaptation of the HPA axis and inflammatory system to a series of physiological challenges (i.e., CPT), are able to discriminate between groups of well-phenotyped individuals with insomnia disorder and healthy control sleepers. We expected that individuals with insomnia are more reactive to a physiological challenge compared to healthy control sleepers, as manifested in a stronger response of HPA (cortisol) and inflammatory (IL-6) markers, and show less HPA and inflammatory adaptation (i.e., response decrease) across the three challenges.

The HPA axis and inflammatory system are tightly regulated, such that inflammatory cytokines activate the HPA axis, and cortisol, in turn, blunts the production of cytokines by monocytes, one of the major producer of inflammatory markers (Chrousos, 1995, Wilder, 1995). One potential mechanism that could contribute to increased inflammatory reactivity, as well as increased inflammatory markers at rest, is a reduced sensitivity of immune cells to the counter-inflammatory glucocorticoid (GC) signal. Indeed, a reduction in GC sensitivity has been reported in patients suffering from rheumatoid arthritis (RA) or fibromyalgia (Geiss et al., 2012, Quax et al., 2013), as well as in response to acute (e.g., interview) and chronic stressors (e.g., caregiving for a cancer patient) in healthy participants (Miller et al., 2002, Rohleder et al., 2003, Rohleder, 2012, Sauer et al., 1995). Such reductions in GC sensitivity are thought to be responsible for low-grade inflammation frequently reported under these conditions. However, increased GC sensitivity has been reported too, such as in patients with depression, myalgic encephalopathy/chronic fatigue syndrome (ME/CFS), or post-traumatic stress disorder (PTSD; Gaab et al., 2003; Miller et al., 2005; Rohleder et al., 2004), as well as in response to prolonged experimental sleep restriction in healthy participants (Simpson et al., 2016). In the context of insomnia, the sensitivity of immune cells to the GC signal surprisingly has never been assessed despite the association of insomnia disorder with alterations in cytokine and GC production. Thus, the secondary aim of the current study was to investigate whether the interplay between the HPA axis and inflammatory system, as assessed by GC sensitivity of monocytes, differs between individuals with insomnia disorder and healthy control sleepers. We expected that in insomnia, monocytes would be less sensitive to the counter-inflammatory GC signal, underlying the expected exaggerated inflammatory response to a physiological stress challenge as described in the first aim.

## 2 Methods

### 2.1 Study procedures

Participants were recruited via the Beth Israel Deaconess Medical Center sleep clinic, subway postings, internet postings, and flyers. After a preliminary screening via telephone and/or email, eligible participants were invited to come to the Clinical Research Center (CRC) to undergo the informed consent process. The study protocol was approved by the Institutional Review Board of Beth Israel Deaconess Medical Center, and informed written consent was obtained for all participants.

#### 2.1.1 Screening

At the initial screening, participants completed a battery of questionnaires and interviews to help determine eligibility as well as to explore potential psychosocial confounders in stress system reactivity. Questionnaires included the Pittsburgh Sleep Quality Index (Buysse et al., 1989), Pain Catastrophizing Scale (Sullivan et al., 1995), the General Anxiety Disorder Scale (Spitzer et al., 2006), the Perceived Stress Scale (Cohen et al., 1983), the Life Orientation Test (Scheier et al., 1994), the Patient Health Questionnaire-9 (Kroenke et al., 2001) and the Standard Form-36 (Ware et al., 2000). A clinical interview using the Duke Structured Sleep Disorders Intake Interview (Edinger et al., 2011) was given to assist in the diagnosis of insomnia disorder based on DSM-V and to evaluate the presence of other sleep disorders, such as nightmare disorder or circadian rhythm disorder. Moreover, nurses took vital signs and collected a blood sample. Participants in both the insomnia and control group were excluded if the following criteria were present: (a) abnormal blood chemistry, including measures of complete blood counts and differentials, T-cell subsets, liver enzymes, renal and glucose measures, basic coagulation markers and sedimentation rate, and thyroid hormones; (b) toxicology screen positive for substance use; (c) active infection or disease; (d) history of neurological, chronic pain, immune, cardiovascular, liver/kidney, metabolic, or Raynaud’s disease; (e) history of psychiatric disorders in the last 6 months prior to study start; (f) apnea hypopnea index (AHI) of > 15 events/hour or periodic leg movement index (PLMI) of > 10/hour based on polysomnographic screening night; restless legs syndrome, circadian rhythm disorders, and nightmare disorders as determined by diagnostic interview; (g) psychotropic, sleep, or any other medications or herbs interfering with the inflammatory or HPA system in the week prior to study start (except oral contraceptives); (h) In psychotherapy or any other behavioral interventions at study start; (i) pregnant/nursing. Exclusion criteria specific to the control group were self-reported sleep duration of less than 7 or greater than 9 hours/night, sleep onset latency (SOL) of greater than 20 min/night, or wake after sleep onset (WASO) of greater than 20 min/night, as determined by interview. Further, sleep efficiency during the PSG screening night had to be greater than 80%. Inclusion criteria specific to the insomnia group was the presence of insomnia disorder based on DSM-V (American Psychiatric Association, 2013) by clinical diagnostic interview performed by a board-certified sleep physician. For participants who met inclusion criteria, habitual sleep indices were measured by actigraphy and self-report sleep diary over a 2-week recording period between the screening and experimental visit.

#### 2.1.2 Polysomnography sleep and experimental visit

Participants arrived at the CRC in the evening to undergo a medical history/physical and polysomnography (PSG) sleep visit. During the study visit, all food and drink was supplied by the CRC at standardized times and study participants abstained from caffeine or other stimulants. Time in bed was calculated based on participant response during the diagnostic clinical interview to the questions regarding “typical/usual time of lights out and terminal wake time” as well as information from actigraphy to verify habitual time in bed. Discrepancies between diary and actigraphy timings were discussed with patients prior to the PSG recording in order to determine their most typical timings. Screening PSG was scored by a sleep technologist in the morning prior to continuing on to the experimental procedures. An intravenous (iv) line was placed at 1000 for blood drawing starting at 1100. Baseline assessment occurred twice at 1100 and 1130, during which baseline blood was collected to assay inflammatory (IL-6) and HPA markers (cortisol). Following one hour after lunch, the cold pressor test (CPT) was administered 3 times starting at 1300, 1430, and 1600 h. Blood was sampled 20 min and 50 min after hand removal from the cold water bath for each cold pressor test trial (these time points have been shown sensitive to capture increases of cortisol and IL-6 (Edwards et al., 2008, Edwards et al., 2009, Heffner et al., 2012). Prior to, during, and after the cold pressor test series, participants rated the intensity of the cold sensation induced by the CPT. A series of three consecutive CPT challenges was chosen in order to investigate differences in the adaptation of physiological responses to stressors over time between insomnia disorder and healthy control sleepers.

### 2.2 Measurements

#### 2.2.1 Sleep diary

An electronic sleep diary was sent to participants’ email addresses to be filled out every morning and evening over the 2-week recording period (REDCap electronic data capturing system hosted at BIDMC). Diary questions could be completed on a laptop or mobile device. Daily sleep diary data were averaged across the 2-week recording period to determine habitual bedtime, wake time, sleep duration, sleep-onset latency, WASO, sleep efficiency and number of awakenings.

#### 2.2.2 Actigraphy

An actigraph was worn on the non-dominant hand for the 2-week recording period (Philips Respironics Actiwatch® 64; Respironics, Bend, OR, USA). Data were sampled at an epoch length of 30 seconds. Sleep indices were calculated using Actiware 6.0.9 algorithms (Philips Respironics; Ambulatory Monitoring, Ardsley, NY, USA). Daily actigraphy data were averaged across the 2-week recording period to determine habitual bedtime, wake time, sleep duration, sleep-onset latency, WASO, sleep efficiency and number of awakenings.

#### 2.2.3 Polysomnographic recording (PSG)

Sleep was recorded using the Embla system N7000 (Medcare US, Buffalo) on the sleep visit to ensure that participants were free from sleep disorders other than insomnia disorder. The montage followed standard criteria and sleep EEG was manually stage scored on a 30 second epoch basis (American Academy of Sleep Medicine, 2007). We recorded from F3, F4, C3, C4, O1, and O2, referenced to linked mastoids for EEG analysis. Respiratory-related events were measured using nasal cannula, thoracic and abdominal belts, and a pulse oximeter. Scoring of sleep stages, respiratory-related events, and leg movements were performed according to standard criteria (American Academy of Sleep Medicine, 2007).

#### 2.2.4 Physiological stress challenge – cold pressor test (CPT)

The CPT was administered three times during the experimental visit, starting at 1300, 1430 and 1600 h. Throughout the testing period (until 1800), the participant remained in a seated position in a comfortable chair. The CPT was performed using the hand/arm that was not being used for blood sampling in order to prevent interference. For the current study purposes, an inter-test interval of 90 min was chosen to order to allow time to capture response increases of IL-6 and cortisol between trials (Edwards et al., 2008, Edwards et al., 2009, Heffner et al., 2012). For each CPT, participants were asked to insert their hand in a temperature-controlled water bath (Techne® water baths, Bibby Scientific US, Burlington, NJ), kept at 3 degrees C, and instructed to leave their hand in for a duration of at least 1 min. Participants were told that they could withdraw their hand earlier if the sensation was unbearable and were instructed to remove their hand after a maximum of 3 min. The amount of seconds participants were able to tolerate in the bath before removing their hand was considered their level of tolerance. Participants rated the intensity of sensation at 10 sec intervals during and after the testing period (up to 3 min post-testing). Out of the 120 CPTs administered, 13 tests in the insomnia group and 14 tests in the control sleep groups had the maximal hand immersion time of 3 minutes (Chi-Square = 0.01, p = 0.94). Further, 5 tests in the insomnia disorder group had a hand immersion time of less than 60 seconds (average 51.6 ± 2.0 sec), while 4 tests in the control sleep group had a hand immersion time of less than 60 seconds (average 51.8 ± 3.1 sec; Chi-Square = 0.26, p = 0.61 for between-group comparison).

#### 2.2.5 Serum cortisol and plasma IL-6

Blood was drawn 20 and 50 min after hand removal from the cold water bath using an indwelling 18-gauge forearm catheter. IL-6 was measured in plasma in our laboratory using a high sensitivity enzyme immunosorbent assay (ELISA, Quantikine ® HS, R&D Systems, Minneapolis, MN). Samples were measured in duplicates; average intra-assay coefficient of variation was 5.66 ± 0.72%. Cortisol was measured in serum and assayed in the Brigham and Women’s Hospital Research Core Lab using the Access Chemiluminescent Immunoassaay (Beckman Coulter Fullerton, CA).

#### 2.2.6 Glucocorticoid sensitivity of monocytes

GC sensitivity was determined by the capacity of the synthetic glucocorticoid dexamethasone (DEX) to suppress IL-6 expression in monocytes using the 1130 baseline blood sample. Whole blood was stimulated with lipopolysaccharide (LPS) from *Escherichia coli* 0127-B8 (LPS 100 pg/ml, Sigma-Aldrich), and then different concentrations of DEX (0, 12.5, 25, 50, 100, and 200 nM; Sigma- Aldrich) as well as brefeldin A and fluorescence-conjugates antibodies (CD14 APC, CD45 KrO [both Beckman Coulter], IL-6 PE [BD Bioscience]) were added to the samples. Samples were incubated for 4 hours at 37 °C at 5% CO_2_. The samples were analyzed the following day in a Gallios flow cytometer (Beckman Coulter Fullerton, CA, Flow Cytometry Core at BIDMC) using Kaluza software (for details, see Simpson et al., 2016).

### 2.3 Statistical Methods

Power calculations for the outcome measures cortisol reactivity, IL-6 reactivity, and GC sensitivity were based on previously reported findings (Goodin et al., 2012, Heffner et al., 2012, Simpson et al., 2016). A sample size of 20 participants per group was determined to reach 80% probability to detect an effect size of at least Cohen’s d = 0.91 for all three outcome variables. Statistical tests were performed using IBM SPSS statistics software version 23. Independent samples t-tests examined between-group differences in demographic variables and sleep measures; chi-square statistics were used for categorical variables. Partial Pearson’s r controlling for group and time point was used for exploratory correlations between demographic, psychological, sleep and physiological reactivity measures. Strong correlation was determined as r ≥ |0.7|. GLM Mixed Model ANOVA compared cortisol and IL-6 reactivity between insomnia disorder and healthy controls with time point and group as fixed factors and participant number as random factor. These analyzes were performed including time points from all three CPT trials and repeated using time points from the primary CPT trial only in order to gauge initial responsivity. Age, baseline cortisol and baseline IL-6 levels were included as covariates in respective analyses and compound symmetry was determined to be the best fit covariance structure. Glucocorticoid sensitivity was determined via IL-6 dose-response curves for DEX inhibition of LPS-stimulated IL-6 expression and were analyzed by GLM Mixed Model ANOVA with group and concentration of DEX as fixed factors and participant number as random factor. Because baseline levels were used as a covariate, significance of interaction as well as group effects were considered appropriate for follow-up post hoc testing of single time points. Differences between single time points were determined by GLM parameter estimates. In order to determine the magnitude of the influence of insomnia disorder on outcome measures of reactivity and sensitivity, effect sizes (ES) were calculated for the statistical effects of the first CPT and of GC sensitivity at the lowest DEX concentration. Tables and figures present means and standard error of means (SEM). An alpha value of p ≤ 0.05 was considered significant; an alpha value of p ≤ 0.10 was considered a trend towards significance.

## 3 Results

As indicated in Table 1, there were no significant differences in terms of sex, BMI, or race between Insomnia Disorder (ID) and Control groups. Mean age was slightly higher in the insomnia group and was therefore used as a covariate in mixed model analyses. Four participants in the insomnia group reported contraceptive use, compared to six control sleep participants (p = 0.51).

**Table 1:** Demographics and psychological differences between insomnia disorder and controls.

	Insomnia disorder	Healthy controls	Statistics
N (males)	20 (2)	20 (2)	–
Age (range)	18–49 (30 ± 2.5)	18–47 (26 ± 1.4)	t= 1.69, p= 0.10
BMI	23.00 ± 3.19	24.15 ± 3.19	t= 1.28, p= 0.21
			
Race	White: 13	White: 10	χ 2 = 6.72, p = 0.15
	Black: 0	Black: 3	
	Asian: 3	Asian: 6	
	Other: 4	Other: 1	
			
Ethnicity	Hispanic: 2	Hispanic: 1	χ 2 = 1.77, p = 0.41
	Non-Hispanic: 11	Non-Hispanic:15	
	Non-reporting: 7	Non-reporting: 4	
Pain Catastrophizing Scale: Global	17.47 ± 7.84	9.59 ± 5.90	t = 3.31, p = 0.002*
Pain Catastrophizing Scale: Rumination	7.94 ± 3.65	4.76 ± 3.38	t = 2.63, p = 0.013*
Pain Catastrophizing Scale: Magnification	3.53 ± 2.15	2.12 ± 1.58	t = 2.18, p = 0.037*
Pain Catastrophizing Scale: Helplessness	6.06 ± 3.07	2.71 ± 2.54	t;= 3.47, p = 0.002*
General Anxiety Disorder Scale	4.89 ± 4.06	1.05 ± 1.88	t = 3.81, p = 0.001*
Perceived Stress Scale	23.86 ± 4.31	22.35 ± 3.20	t = 1.17, p = 0.25
Life Orientation Test	16.88 ± 3.00	19.47 ± 3.25	t = 2.47, p = 0.018*
Patient Health Questionnaire	5.00 ± 3.09	0.50 ± 1.04	t = 6.76, p <0.001**
Patient Health Questionnaire (with sleep item excluded)	3.25 ± 2.29	0.50 ± 1.04	t = 4.71, p <0.001**
Standard Form-36: Physical Functioning	29.59 ±0.87	29.71 ± 0.90	t = 0.44, p = 0.66
Standard Form-36: Role Physical	7.39 ± 1.24	8.00 ± 0.00	t = 2.26, p = 0.03*
Standard Form-36: Bodily Pain	3.44 ± 1.46	2.57 ± 0.68	t = 2.45, p = 0.019*
Standard Form-36: General Health	13.67 ± 1.97	14.40 ± 1.39	t = 1.34, p = 0.19
Standard Form-36: Vitality	14.44 ± 2.20	16.10 ± 1.73	t = 2.62, p = 0.013*
Standard Form-36: Social Functioning	6.07 ± 0.70	6.00 ± 0.32	t = 0.38, p = 0.71
Standard Form-36: Role Emotional	5.00 ± 1.24	5.81 ± 0.68	t = 2.58, p = 0.014*
Standard Form-36: Mental Health	20.72 ± 1.93	21.43 ± 1.53	t = 1.27, p = 0.21

* p≤0.05.

** p≤0.001.

Participants with insomnia catastrophized pain more than controls on all sub measures (Rumination, Magnification and Helplessness) of the Pain Catastrophizing Scale. Moreover, ID participants reported more general anxiety (GAD) and depression symptom severity (PHQ), less optimism (LOT), greater role limitations due to physical health (SF-36: RP) and emotional problems (SF-36 RE), more bodily pain (SF-36:BP) and less energy (SF:36 V) than Controls (all p < 0.05, see Table 1). Exploratory analysis indicated that none of the demographic or psychological measures were strongly correlated with cortisol or IL-6 baseline or reactivity or GC sensitivity (*data not shown*). Intensity ratings following CPT trials were similar between groups (group: F = 0.18, p = 0.67; group-by-time: F = 2.06, p = 0.14). Tolerance duration following CPT trials showed a group-by-time effect such that average tolerance duration increased with subsequent trials in the Control group but decreased in the ID group (group: F = 0.009, p = 0.92; group-by-time: F = 3.93, p = 0.03). Controlling for tolerance duration only marginally affected between-group differences in cortisol reactivity, IL-6 reactivity, and GC sensitivity (*data not shown*).

### 3.1 Sleep differences between insomnia disorder and controls

Habitual sleep (as assessed by actigraphy and sleep diary) and PSG sleep variables are summarized in Table 2, Table 3, respectively. The majority of participants in the ID group reported suffering from insomnia for over 5 years (see Table 2). ID participants had significantly higher scores on the Pittsburgh Sleep Quality Index (indicating worse sleep quality) and reported shorter sleep duration, longer sleep latency (SL), longer wake after sleep onset (WASO) and a greater number of nighttime awakenings on the daily diary. Control participants objectively slept ~ 40 minutes longer than ID both habitually (actigraphy sleep duration; see Table 2) and on the night prior to testing (PSG sleep duration; see Table 3). Objective measures of sleep efficiency, number of awakenings, SL, WASO or bedtime were not significantly different between groups (Table 2, Table 3, all p > 0.05). Exploratory analysis indicated that no sleep measures were strongly correlated with cortisol or IL-6 baseline or reactivity or GC sensitivity (*data not shown*).

**Table 2:** Habitual Sleep Differences between Insomnia Disorder and Controls.

		Insomnia disorder	Healthy controls	Statistics
Insomnia Duration		< 1 year:3	–	–
		1–5 years: 6		
		> 5 years: 11		
Pittsburgh Sleep Quality Index		10.05 ± 2.74	1.86 ± 1.35	t = 12.18, p < 0.001**
				
Diary	Bedtime	2332 ± 0117	2407 ± 0047	t = 1.69, p = 0.10
	Wake time	0646 ± 0113	0718 ± 0219	t = 0.91, p = 0.36
	Total Sleep Time, in minutes	393 ± 54	438 ± 34	t = 5.22, p < 0.001**
	Sleep Latency, in minutes	49 ± 65	14 ± 9	t = 2.39, p = 0.022*
	WASO, in minutes	30 ± 23	5 ± 7	t = 4.71, p < 0.001**
	Number of Awakenings	1.92 ± 1.20	0.78 ± 0.56	t = 3.83, p < 0.001**
				
Actigraphy	Bedtime	2323 ± 0128	2355 ± 0104	t = 1.28, p = 0.21
	Wake time	0710 ± 0122	0805 ± 0054	t = 2.45, p = 0.019*
	Total Sleep Time, in minutes	398 ± 57	441 ± 63	t = 2.24, p = 0.031*
	Sleep Efficiency, in percent	83.05 ± 5.42	83.46 ± 6.63	t = 0.21, p = 0.83
	Sleep Latency, in minutes	21 ± 11	22 ± 12	t = 0.38, p = 0.71
	WASO, in minutes	44 ± 19	38 ± 18	t = 1.07, p = 0.29
	Number of Awakenings	34.48 ± 13.64	34.47 ± 13.54	t = 0.003, p = 0.99

* p< 0.05.

** p< 0.001.

**Table 3:** Previous night’s sleep differences between insomnia disorder and controls as measured by polysomnography.

	Insomnia disorder	Healthy controls	Statistics
Total Sleep Time, in minutes	389.12 ± 92.26	439.63 ± 39.88	t = 2.25, p = 0.031*
Sleep Onset Latency, in minutes	36.93 ± 43.56	24.17 ± 28.62	t = 1.09, p = 0.28
Sleep Efficiency, in percent	78.18 ± 23.31	88.72 ± 5.72	t = 1.96, p = 0.057
Wake After Sleep Onset, in minutes	49.89 ± 80.80	31.37 ± 20.00	t = 0.99, p = 0.33
Number of Awakenings	16.85 ± 9.84	19.35 ± 9.02	t = 0.84, p = 0.41
Apnea Hypopnea Index (AHI), events/hour	2.8 ± 1.1	1.6 ± 0.5	t = 0.93, p = 0.13
Periodic Leg Movement Index (PLMI), events/hour	0.5 ± 0.2	0.8 ± 0.2	t = 0.39, p = 0.43

**=p<0.001

* p<0.05.

### 3.2 Cortisol and IL-6: Basal levels and reactivity to a repeated physiological stress challenge

**Fig. 1:**
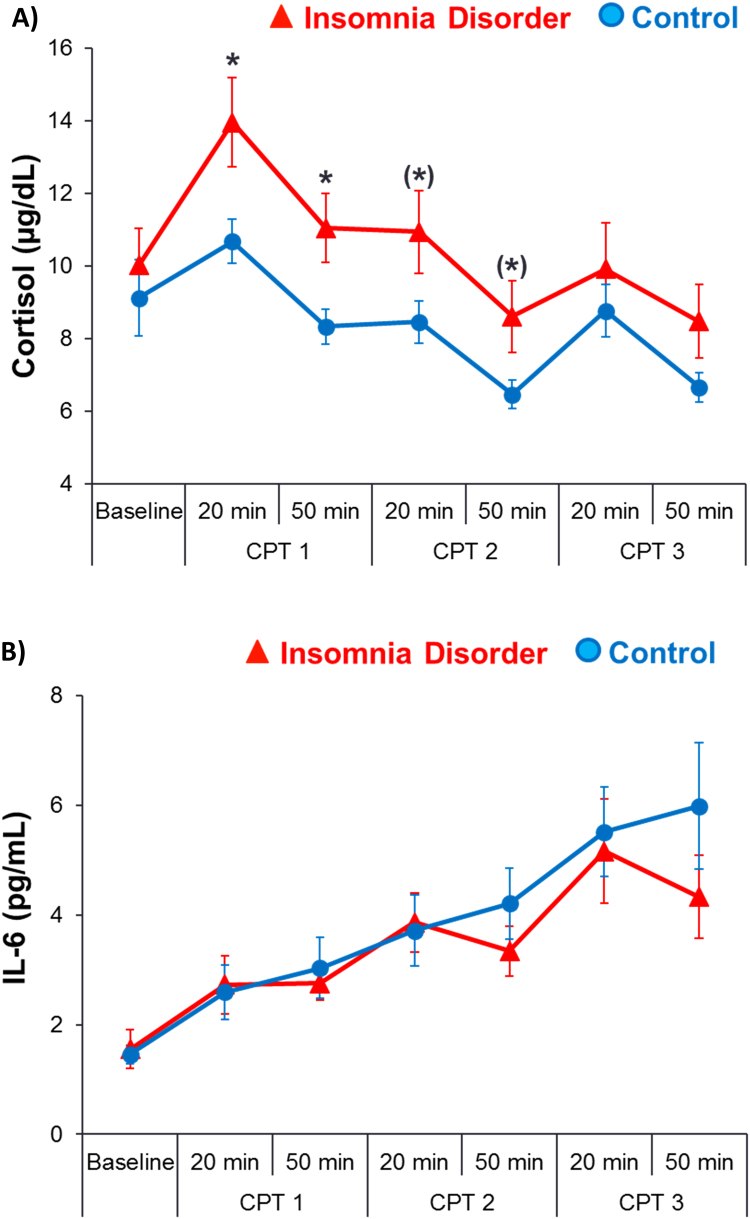
Cortisol and IL-6 Reactivity to a Repeated Physiological Stress Challenge. Cortisol (A) and IL-6 (B) responses to a repeated physiological stressor, i.e., the cold pressor test (CPT) in individuals with insomnia disorder compared to healthy controls. Presented as means ± SEM. (A) cortisol reactivity: P ≤ 0.05 for group effect, controlling for age and baseline levels; (B) IL-6 reactivity: p > 0.05 for group or group by time interaction effect, controlling for age and baseline levels. *p ≤ 0.05 and (*)p ≤ 0.10 for single time point comparisons.

There were no significant between-group differences in 1100 or 1130 levels of cortisol or IL-6 nor in averages between these two time points, which served as baseline measures (all p > 0.36). As shown in Fig. 1A, there was a significant overall group effect for cortisol reactivity to the repeated CPT (F = 4.12, p = 0.05), while the group-by-time interaction effect was not significant (F = 1.18, p = 0.32). Cortisol reactivity in insomnia was higher following the initial CPT trial compared to controls (20 min: p = 0.01, ES = 0.71; 50 min: p = 0.04, ES = 0.75). There was also a trend for greater cortisol reactivity in insomnia participants for the second trial (20 min: p = 0.06; 50 min: p = 0.10) but not the third trial (20 min: p = 0.36; 50 min: p = 0.17).

As shown in Fig. 1B, there was no significant group (F = 1.25, p = 0.27), or group-by-time interaction effect (F = 0.93, p = 0.46) in IL-6 reactivity to the repeated CPT. There were also no significant group (F=0.25, p=0.62), or group-by-time interaction effects (F = 0.37, p = 0.69) in IL-6 reactivity just with respect to the primary CPT trial (20 min: p > 0.05, ES = 0.08; 50 min: p > 0.05, ES = 0.06), or subsequent CPT trials (all p>0.05).

### 3.3 Glucocorticoid sensitivity in insomnia disorder and controls

**Fig. 2:**
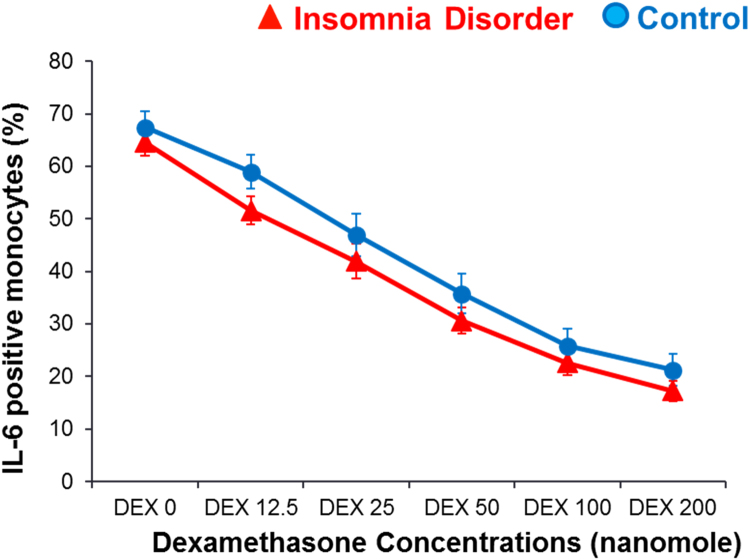
Glucocorticoid Sensitivity in Insomnia Disorder and Controls. GC sensitivity determined by the ability of dexamethasone (DEX) to suppress IL-6 expression in monocytes in insomnia disorder and healthy controls. Higher IL-6 suppression by DEX indicates higher GC sensitivity. Presented as means ± SEM. P > 0.05 for group or interaction effect, controlling for age and baseline IL-6 expression.

Fig. 2 presents the GC sensitivity determined by the ability of dexamethasone (DEX) to suppress IL-6 expression in monocytes across different concentrations of DEX in ID and Control participants. As expected, IL-6 positive monocytes decreased with increasing DEX concentrations (F = 348.79. p < 0.001 for concentration effect). Mixed model analysis indicated no significant group (F = 1.32, p = 0.26) or group-by-concentration interaction effects (F = 0.97, p = 0.45, ES = 0.54 at DEX concentration of 12.5) in the ability to suppress IL-6. IL-6 positive monocytes at baseline (without DEX), did not differ between groups when stimulated with LPS (t = 1.01, p = 0.32, ES = 0.24).

Excluding the two men from the analysis did not change results for cortisol reactivity (p = 0.045 for group effect), IL-6 reactivity (p = 0.71 for group effect), or GC sensitivity (p = 0.44 for group effect). Co-varying for time of hand immersion did not substantively alter the results.

## 4 Discussion

The current study investigated HPA axis and inflammatory responses to a repeated physiological stressor and basal differences in GC sensitivity in individuals with insomnia disorder and healthy controls. Glucocorticoid sensitivity as well as HPA and inflammatory reactivity to stressful challenges have not hitherto been explored as potential indices of physiological dysregulation in insomnia disorder. This investigation therefore represents a first step towards understanding the role of these physiological components in insomnia disorder.

Cortisol reactivity in response to a repeated physiological stressor was greater in insomnia disorder, while IL-6 reactivity in insomnia disorder was similar to control participants. The overall effect of a greater cortisol reactivity in insomnia was mainly due to responses following the first, initial CPT (Fig. 1A). Cortisol responses still trended towards significance following the second CPT, but did not differ any longer from responses of control participants following the last CPT. This finding suggests that individuals with insomnia disorder have an exaggerated response to a novel stressor, but are able to adapt (i.e., show a response decrease) to stressor repetition to the same extent as healthy sleepers. While lack of adaption to the same stressor has been hypothesized as a mechanism contributing to adverse health outcomes (McEwen, 1998), current findings do not support that stress adaptation is affected in young individuals with insomnia disorder. However, findings of a stronger cortisol response following the first stressor (i.e., the first CPT) suggest a HPA hyper-reactivity to novel challenges. Increased HPA reactivity following a physiological CPT challenge has been previously found in a sample reporting poor sleep compared to those reporting good sleep (Goodin et al., 2012), as well as following the pharmacological combined DEX/CRH challenge test (Hori et al., 2011). However, self-reported poor sleep quality has not always been found to potentiate HPA reactivity in studies using psychosocial or cognitive stressors (for review, see (van Dalfsen and Markus, 2018), and in a recent study in individuals fulfilling diagnostic criteria of insomnia, cortisol reactivity in response to an electric shock stressor was not different from healthy sleepers (Gehrman et al., 2016). Factors that have been suggested to contribute to variability in study findings are the type of experimental stressor (i.e., physiological, psychosocial, cognitive), age, and sex (see (van Dalfsen and Markus, 2018). In contrast to exaggerated cortisol reactivity to the physiological CPT challenge in insomnia disorder, the current study did not reveal changes in inflammatory (IL-6) reactivity, contrasting with previous findings of increased IL-6 reactivity to psychosocial and cognitive stressors in individuals with poor sleep (Heffner et al., 2012, Prather et al., 2014). It is possible that an age-dependent effect contributes to these discrepant findings. While the current study sample was of young age, previous studies reporting increased IL-6 reactivity included older men and postmenopausal women age 50 or older (Heffner et al., 2012, Prather et al., 2014). Thus, changes in inflammatory reactivity may become evident when insomnia disorder occurs with advanced age, but not in young adults.

Beyond HPA and inflammatory reactivity, we also investigated the interplay between HPA axis and the inflammatory system by assessing the sensitivity of monocytes, the major producers of IL-6, to the counter-inflammatory cortisol signal. To our knowledge, this is the first study to examine GC sensitivity in individuals with insomnia. ID participants’ basal GC sensitivity of monocytes did not appear to be disrupted compared to healthy control participants. Interestingly, a recent study using the DEX/CRH suppression/stimulation test reported no differences between individuals with insomnia and healthy controls, indicating normal feedback sensitivity of the HPA system (Lattova et al., 2011). These findings suggest that HPA/inflammatory interactions at the cell level appear to be normal in individuals with insomnia disorder. In the context of deficient sleep, a recent study on the effects of experimental prolonged sleep restriction over a 3-week long period in healthy participants found an increased GC sensitivity in monocytes (Simpson et al., 2016), and despite this increase, IL-6 expression in monocytes was still upregulated in response to sleep restriction. Increased, but also reduced GC sensitivity has been reported in disorders were sleep disturbances are common, including mental health disorders (depression, PTSD), pain-related disorders (RA, fibromyalgia), and ME/CFS (Gaab et al., 2003, Geiss et al., 2012, Miller et al., 2005, Quax et al., 2013, Rohleder et al., 2004). As discussed above, abnormalities may become evident in the combination of insomnia disorder with advanced age or duration of the disorder. Of note, the absence of GC sensitivity changes in insomnia disorder in the current study is consistent with unchanged basal levels of cortisol and IL-6, as well as unchanged inflammatory reactivity to challenge in this young study sample with insomnia disorder.

Insomnia participants in this study objectively slept for shorter duration than controls both habitually and during the night prior to experimental testing. It has been suggested that objective sleep disturbances are required in order to observe the related biological impact of insomnia (Floam et al., 2015, Vgontzas et al., 2013). However, the differentiation between short duration and normal sleep duration is often an average cutoff of 6 hours sleep per night. In our insomnia population, the average sleep duration was over 6 hours for all but three participants. It is therefore also possible that, with the exception of an increased HPA reactivity to challenge, measures of basal levels of cortisol, IL-6, and GC sensitivity, as well as inflammatory reactivity to challenge did not differ between groups because objective sleep duration was not sufficiently shortened.

One limitation of the current study is that increases in cortisol and IL-6 levels over the course of the CPT trials were not compared to circadian changes in these markers in the absence of physiological stressors. As such, it is difficult to ascertain whether there is an actual inflammatory response to the CPT. Furthermore, the time interval between the repeated CPT series was 90 min only. While increases of cortisol and IL-6 have been reported within this time window, their recovery to baseline values has not been well studied. This is in particular true for inflammatory recovery. As evident in the current study, the IL-6 response is not recovering within the 90-min interval post-stressor, making it difficult to investigate inflammatory habituation. Thus, HPA and inflammatory responses to the first CPT may have prevented an accurate measure of subsequent CPT responses. Spacing challenges by longer time intervals, for example, 24 hours, would allow for sufficient recovery and the ability to control for circadian influences. In addition, increasing the frequency of blood sampling following the physiological challenges would increase precision in determining slope and peak values of measures.

To conclude, resting levels of cortisol and IL-6, and their interplay as assessed by GC sensitivity on the cell level were unaltered in individuals with insomnia disorder compared to healthy control participants. However, insomnia disorder potentiates HPA (but not inflammatory) reactivity following the repeated exposure to the same physiological stressor. While HPA adaptation to stress repetition was unaltered, reactivity to the initial stressor was much stronger. Such HPA over-reactivity to the many stressors in daily life may constitute one pathway through which insomnia disorder may contribute to adverse health consequences.

## Declarations of interest

None.

## Conflicts of Interest

There are no conflicts of interest to disclose.
